# Leveraging digital medication adherence technologies to enhance sustainability of European health systems: ENABLE’s key recommendations

**DOI:** 10.1016/j.lanepe.2024.101164

**Published:** 2024-12-03

**Authors:** Job F.M. van Boven, Alexandra L. Dima, Björn Wettermark, Ines Potočnjak, Tamás Ágh, ENABLE collaborators, Emma Aarnio, Maria Achterbosch, Tamás Ágh, Nilay Aksoy, Martina Bago, Pilar Barnestein-Fonseca, Noemi Bitterman, Job F.M. van Boven, Michel Burnier, Edel Burton, Theodosia Charitou, Maria Cordina, Tinne Dilles, Alexandra L. Dima, Klemen Dovc, Marie Ekenberg, Monique Elseviers, Válter R. Fonseca, Sabina de Geest, Cristina M. Ghiciuc, Catherine Goetzinger, Hanna Gottlieb, Anne-Gerd Granas, João Gregório, Gaye Hafez, Kjeld Hansen, Frederik Haupenthal, Rob Heerdink, Maria Teresa Herdeiro, Mickaël Hiligsmann, Dalma Hosszú, Cristina Jacome, Fatjona Kamberi, Maria Kamusheva, Anthony Karageorgos, Przemyslaw Kardas, Nataliia Khanyk, Sandrine Lavalle, Francisca Leiva Fernández, Carlotta Lunghi, Enrica Menditto, Jovan Mihajlovic, Iva Mucalo, Sara Mucherino, Urska Nabergoj Makovec, Anna Oleárová, Maja Ortner Hadžiabdić, Christos Petrou, Ana Tomas Petrovic, Guenka Petrova, Hilary Pinnock, Elisabetta Poluzzi, Ines Potočnjak, Miriam Qvarnström, Pamela Rackow, Janette Ribaut, Fatima Roque, Laura J. Sahm, Marie Paule Schneider, Katarina Smilkov, Dins Smits, Ivana Tadic, Indrė Trečiokienė, Ioanna Tsiligianni, Katia Vermeire, Marcia Vervloet, Jiří Vlček, Shlomo Vinker, Daisy Volmer, Bernard Vrijens, John Weinman, Björn Wettermark

**Affiliations:** a Department of Clinical Pharmacy & Pharmacology, Medication Adherence Expertise Center Of the northern Netherlands (MAECON), University Medical Center Groningen, University of Groningen, Groningen, the Netherlands; b Avedis Donabedian Research Institute (FAD) - Universitat Autònoma de Barcelona (UAB), C / Provença 293, Barcelona, Spain; c Health Technology Assessment in Primary Care and Mental Health (PRISMA), Institut de Recerca Sant Joan de Déu, Santa Rosa 39-57, Esplugues de Llobregat 08950, Spain; d Consortium “Centro de Investigación Biomédica en Red” Epidemiology and Public Health (CIBERESP), Madrid, Spain; e Department of Pharmacy, Faculty of Pharmacy, Uppsala University, Box 580, Uppsala 751 23, Sweden; f Sestre Milosrdnice University Hospital Center, Zagreb, Croatia; g School of Medicine, Catholic University of Croatia, Zagreb, Croatia; h Syreon Research Institute, Budapest, Hungary

European health systems are under pressure as a result of ageing populations, staff shortages and economic constraints. Therefore, digitalization of care processes, e.g., supporting patients’ medication use, is being increasingly explored.^1^ The Organisation for Economic Co-operation and Development highlights that poor medication adherence is associated with 200,000 premature deaths and €125 billion of wasted costs in Europe annually, positioning this topic as a key challenge for health system efficiency.^2^ Digital patient support could be of help here. Notably, the uptake of digital tools to support medication use across Europe was accelerated by the COVID-19 pandemic.^3^ These digital adherence technologies, such as digital pill dispensers, smart inhalers and electronic blisters, are technically promising and their data may be valuable to inform the European Health Data Space, yet implementation challenges related to privacy and reimbursement persist.^4^

In 2020, the European Cooperation in Science and Technology (COST) funded the 4-year European Network to Advance Best practices and technoLogy on medication adherencE (ENABLE) COST Action, encompassing around 250 collaborators from 40 countries.^5^ Ever since, ENABLE has benchmarked the state of implementation of digital adherence technologies across Europe. Amongst others, current barriers and facilitators for medication adherence management by healthcare professionals in 37 European countries were mapped, a freely accessible repository of digital adherence technologies was developed (https://enableadherence.eu/enable-repository-matech/) and relevant reimbursement pathways were explored.

In August 2024, ENABLE’s final dissemination event “World Adherence Forum” took place in Brussels, with presence from many relevant organisations and stakeholders, such as the World Health Organisation (WHO), patient organisations (e.g., the European Lung Foundation [ELF]), healthcare professionals (e.g., the World Organization of Family Doctors [WONCA], European Association for the Study of Diabetes [EASD]), medtech and pharmaceutical companies. During the meeting, ENABLE’s key findings were discussed with stakeholders and placed in the broader context of Europe’s health system resilience. Following these discussions, key recommendations were formulated (Table 1).

**Table 1:** Key recommendations regarding implementation of digital adherence technologies in Europe from COST Action ENABLE.

**Acknowledge**
• Around 50% of medication is not taken as prescribed • Medication non-adherence is associated with an estimated 200,000 deaths and a financial burden of €125 billion annually in Europe • Enhancing medication adherence improves clinical outcomes, while reducing the financial and environmental burden on health systems
**Inform**
• Adoption of digital technologies can effectively support medication adherence and improve patients’ health outcomes • Innovative digital technologies foster communication and collaboration among healthcare professionals and patients, and should be made more accessible
**Incentivise**
• Increased investment and incentives to accelerate sustainable, cost-effective implementation of digital adherence technologies in health systems are necessary • Digital adherence technology should be an integral part of clinical trials to support efficient drug development
**Steer & support**
• Data-driven European strategies to improve medication adherence are essential • Reimbursement of effective digital adherence technology is critical to unlock their full potential of making health systems more efficient

First, it is important to acknowledge the wide prevalence and impact of medication nonadherence, but also the significant gains when adequately addressing it. Second, stakeholders and policy makers should be informed on the beneficial effects of digital technologies to support adherence. Third, incentives should be provided to accelerate the uptake of digital technologies in health systems and clinical trials. Finally, European data-driven monitoring, implementation strategies and reimbursement are required.

With these recommendations, we urgently call for European policy makers to invest more resources in making digital adherence technologies available more widely, thereby enhancing patients’ health outcomes, and strengthening the sustainability and efficiency of European health systems.

## Contributors

JFM van Boven was Chair of the ENABLE consortium and wrote the first draft of this Correspondence, other authors were ENABLE Working Group leads and provided input and approved the final version. ENABLE collaborators contributed to drafting the key ENABLE recommendations. ENABLE collaborators included ENABLE consortium members and participants in the World Adherence Forum. The World Adherence Forum was the place where the key ENABLE recommendations were discussed and formulated and was attended by active ENABLE consortium members as well as external stakeholders.

## Declaration of interests

JFM van Boven reports being Chair of COST Action ENABLE and his institution (UMCG) has received grants and consultancy funding from companies working in the field of medication adherence. T Ágh reports funding for attending meetings of COST Action CA19132 “ENABLE” and that his institution (Syreon Research Institute) received grants from pharmaceutical industries and NGOs. I Potočnjak reports funding for attending meetings of COST Action CA19132 “ENABLE”.
